# Association between systemic inflammation response index and chronic kidney disease: a population-based study

**DOI:** 10.3389/fendo.2024.1329256

**Published:** 2024-02-22

**Authors:** Xiaowan Li, Lan Cui, Hongyang Xu

**Affiliations:** Department of Critical Care Medicine, The Affiliated Wuxi People’s Hospital of Nanjing Medical University, Wuxi, China

**Keywords:** systemic inflammation response index, chronic kidney disease, albuminuria, estimated glomerular filtration rate, cross-sectional study

## Abstract

**Introduction:**

Our objective was to explore the potential link between systemic inflammation response index (SIRI) and chronic kidney disease (CKD).

**Methods:**

The data used in this study came from the National Health and Nutrition Examination Survey (NHANES), which gathers data between 1999 and 2020. CKD was diagnosed based on the low estimated glomerular filtration rate (eGFR) of less than 60 mL/min/1.73 m^2^ or albuminuria (urinary albumin-to-creatinine ratio (ACR) of more than 30 mg/g). Using generalized additive models and weighted multivariable logistic regression, the independent relationships between SIRI and other inflammatory biomarkers (systemic immune-inflammation index (SII), monocyte/high-density lipoprotein ratio (MHR), neutrophil/high-density lipoprotein ratio (NHR), platelet/high-density lipoprotein ratio (PHR), and lymphocyte/high-density lipoprotein ratio (LHR)) with CKD, albuminuria, and low-eGFR were examined.

**Results:**

Among the recruited 41,089 participants, males accounted for 49.77% of the total. Low-eGFR, albuminuria, and CKD were prevalent in 8.30%, 12.16%, and 17.68% of people, respectively. SIRI and CKD were shown to be positively correlated in the study (OR = 1.24; 95% CI: 1.19, 1.30). Furthermore, a nonlinear correlation was discovered between SIRI and CKD. SIRI and CKD are both positively correlated on the two sides of the breakpoint (SIRI = 2.04). Moreover, increased SIRI levels were associated with greater prevalences of low-eGFR and albuminuria (albuminuria: OR = 1.27; 95% CI: 1.21, 1.32; low-eGFR: OR = 1.11; 95% CI: 1.05, 1.18). ROC analysis demonstrated that, compared to other inflammatory indices (SII, NHR, LHR, MHR, and PHR), SIRI exhibited superior discriminative ability and accuracy in predicting CKD, albuminuria, and low-eGFR.

**Discussion:**

When predicting CKD, albuminuria, and low-eGFR, SIRI may show up as a superior inflammatory biomarker when compared to other inflammatory biomarkers (SII, NHR, LHR, MHR, and PHR). American adults with elevated levels of SIRI, SII, NHR, MHR, and PHR should be attentive to the potential risks to their kidney health.

## 1 Introduction

An estimated 10% of the global population has chronic kidney disease (CKD), which is associated with a major economic and public health burden (1). The United States (US) continues to have one of the highest rates of end-stage renal disease (ESRD) worldwide (2). The optimal management of CKD and its prevention have emerged as crucial public health issues due to its high prevalence, prevalence, and healthcare costs. Inflammation, obesity, diabetes, hypertension, and cardiovascular diseases (CVD) are all risk factors for CKD (3). Inflammation, as an increasingly prominent modifiable risk factor, plays a vital role in establishing effective treatment strategies to prevent the onset and progression of CKD in clinical practice.

One new inflammatory biomarker that is predictive for a number of diseases is the systemic inflammation response index (SIRI), which is derived from counts of neutrophils, monocytes, and lymphocytes. Previous studies have found that SIRI can predict the severity and development of acute pancreatitis (AP) (4). For hepatocellular carcinoma (HCC) patients undergoing systemic therapy, SIRI is a standalone prognostic factor (5). The study by Xia et al. concentrated on the positive correlation between SIRI with cardiovascular mortality and all-cause mortality in American people (6).

Prior research has indicated a close relationship between inflammation and CKD. An association was shown between elevated albuminuria prevalence and elevated values of the systemic immune inflammation index (SII) by Qin et al. (7). One biomarker that helps forecast how CKD will progress is the neutrophil-to-lymphocyte ratio (NLR) (8). Monocyte-to-lymphocyte ratio (MLR) and the risk of 90-day all-cause death in patients with type 2 diabetes (T2DM) and diabetic kidney disease (DKD) were found to be significantly correlated by Qiu’s research (9). Monocyte/high-density lipoprotein ratio (MHR) may be a biomarker for predicting DKD (10). However, there has been no previous research investigating the association between SIRI and CKD.

Determining the relationship between SIRI and CKD is the goal of this study, which uses data from the National Health and Nutrition Examination Survey (NHANES).

## 2 Materials and methods

### 2.1 Survey description

To get a representative sample of the non-institutionalized, civilian U.S. population, NHANES was conducted twice a year in the country. The US population’s health and nutritional condition were tracked over time by the NHANES using a sophisticated, multistage probability sampling design. The National Center for Health Statistics (NCHS) Ethics Review Board approved the study, and everyone who participated in NHANES gave their informed consent. NHANES was carried out by the Centers for Disease Control and Prevention (CDC).

### 2.2 Study population

**Figure 1:**
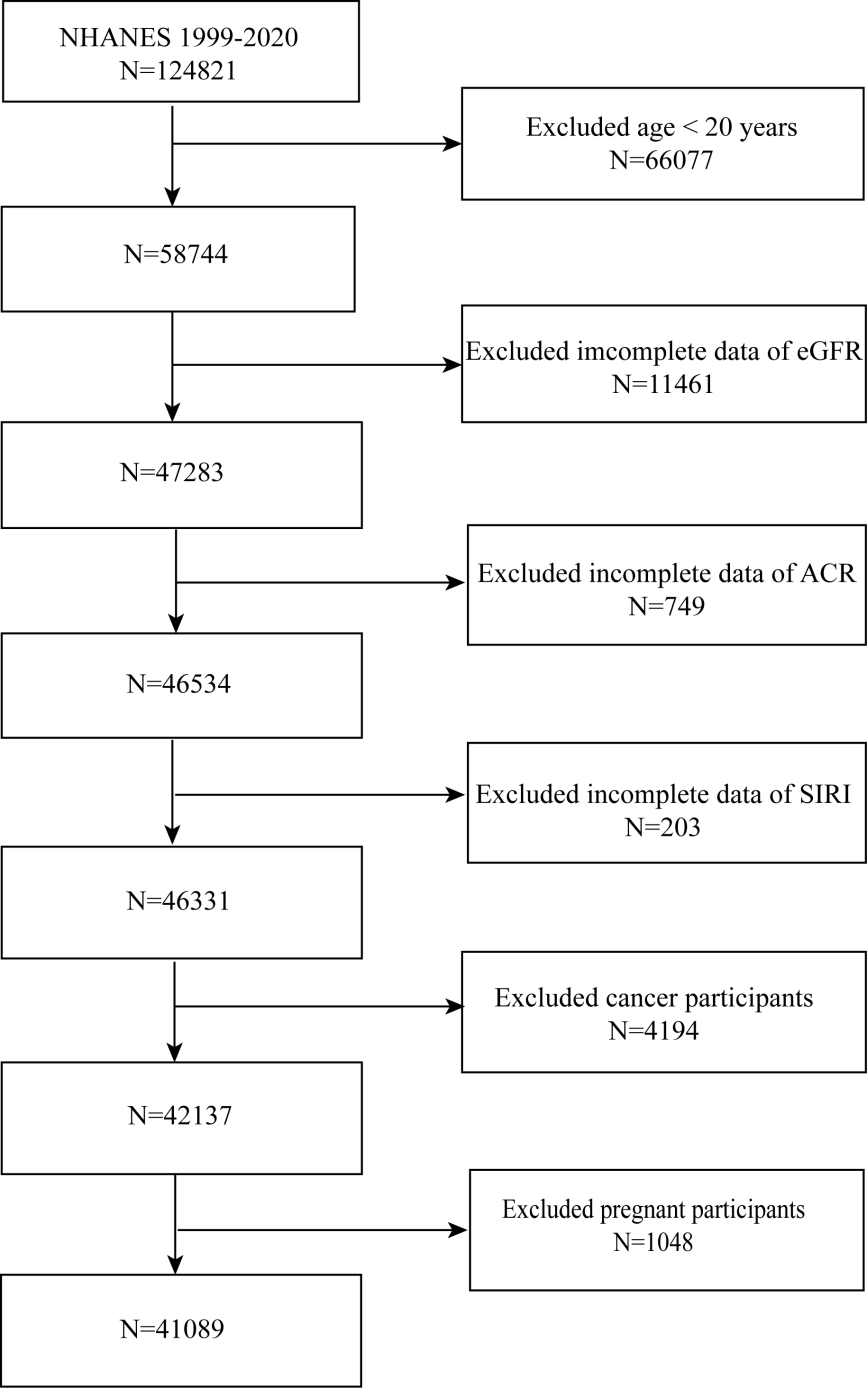
Flowchart of the sample selection from NHANES 1999–2020.

NHANES 1999-2020 individuals were gathered for this investigation. We concluded that 41,089 people were suitable after excluding patients with cancer (n = 1,285), age < 20 (n = 48,975), or pregnancy (n = 220), as well as those without the albumin-to-creatinine ratio (ACR) (n = 8,506), estimated glomerular filtration rate (eGFR) (n = 16,013), and SIRI (n = 1,125) (**Figure 1**).

### 2.3 Definition of SIRI and CKD

Blood cell count measurements were conducted using the Beckman Coulter MAXM automated analytical instrument (Beckman Coulter Inc.). Counts for lymphocytes, neutrophils, monocytes, and platelets were expressed in units of ×10^3^ cells/μl. In our study, SIRI was considered as the primary exposure variable. Neutrophil count × monocyte count/lymphocyte count is how SIRI is computed (11). To further explore the connection between SIRI and CKD, we also examined the link between other inflammatory biomarkers and renal function. These additional inflammation biomarkers included SII, MHR, neutrophil/high-density lipoprotein ratio (NHR), platelet/high-density lipoprotein ratio (PHR), and lymphocyte/high-density lipoprotein ratio (LHR). SII = platelet counts × neutrophil counts/lymphocyte counts, NHR = neutrophil counts/high-density lipoprotein cholesterol (HDL-C) (mmol/L), MHR = monocyte counts/HDL-C, LHR = lymphocyte counts/HDL-C, and PHR = platelet counts/HDL-C are the formulas for these inflammatory indicators (12). To measure HDL-C levels, chemical analyzers Roche Cobas 6000 and Roche modular P were utilized.

To diagnose CKD, one of two conditions must exist albuminuria or an eGFR of less than 60 mL/min/1.73 m^2^ (13). In 2009, the equation for standardized creatinine developed by the Chronic Kidney Disease Epidemiology Collaboration (CKD-EPI) was used to determine eGFR (14). ACR ≥ 30 mg/g was utilized to characterize albuminuria. In our investigation, the outcome variables were albuminuria, low-eGFR, and CKD.

### 2.4 Selection of covariates

Our study employed a set of covariates to control for potential confounding factors. These confounders included age, race, sex, marital status, family income to poverty ratio (PIR), and educational level, among other demographic factors. We also included a number of laboratory and anthropometric factors, including CVD, alcohol consumption, smoking status, total cholesterol (TC), triglycerides, aspartate aminotransferase (AST), alanine aminotransferase (ALT), blood uric acid, serum phosphorus, and body mass index (BMI) (7, 15–17).

The word “hypertension” in this study refers to three different things. At first, respondents self-reported having high blood pressure in response to the survey question “Ever told you had hypertension.” Examining if average blood pressure surpassed 130/80 mmHg diastolic or systolic values was the second component (18). The last phase used the item “taking hypertension prescription” program to identify people who had hypertension. Likewise, this study’s definition of diabetes is divided into three sections. Diabetes self-reporting is covered in the first section, and using insulin or prescription drugs is covered in the second. Lastly, hemoglobin A1c (HbA1c) (%) > 6.5 and fasting blood sugar levels (mmol/l) ≥ 7.0 were used to identify those with diabetes. Visit the website www.cdc.gov/nchs/nhanes/ for further information.

### 2.5 Statistical analysis

When doing the statistical analyses, which took into account intricate multistage cluster surveys and made use of the proper NHANES sampling weights, the CDC guidelines were adhered to. When presenting categorical variables by proportions, standard error (SE) were used to represent continuous variables. To analyze the differences between participants categorized by SIRI tertiles, either a weighted Student’s t-test or a weighted chi-square test was used (for continuous variables). The correlation between SIRI and CKD was examined in three distinct models using multivariable logistic regression. Covariates were not adjusted in Model 1. Race, age, and sex were adjusted in Model 2. The variables sex, age, race, education level, BMI, alcohol consumption, smoking status, TC, triglycerides, AST, ALT, PIR, CVD, blood uric acid, serum phosphorus, marital status, diabetes, and hypertension were all adjusted for in Model 3. We tested the robustness of our results by doing a sensitivity analysis using SIRI converted from a continuous variable to a categorical variable (tertiles). Nonlinear relationships were addressed using generalized additive models (GAM) and smooth curve fitting. Utilizing the log-likelihood ratio test, we compared the segmented regression model—a two-segment linear regression model fitted to each interval—against the non-segmented model, or a one-line model. This allowed us to further investigate threshold effects. To locate breakpoints, we used a two-step recursive technique. Subgroup analyses were conducted using stratified multivariable logistic regression models, with stratification based on sex, age, BMI, hypertension, CVD, and diabetes (7, 19). These stratification factors were considered potential effect modifiers to assess heterogeneity in the correlations between subgroups. Additionally, the predictive efficacy of NLR and other inflammatory biomarkers (SII, NHR, LHR, MHR, and PHR) was assessed through the examination of area under the curve (AUC) values and the use of receiver operating characteristic (ROC) curves (20, 21). For categorical variables, mode imputation was utilized to resolve missing values, whereas median imputation was applied to continuous variables. For all of our statistical analyses, we utilized the Empower software suite and R version 4.1.3. A two-tailed *p*-value < 0.05 was used to determine statistical significance.

## 3 Results

### 3.1 Participants characteristics at baseline

Among the 41,089 participants in our analysis, 48.26% of them were men and 51.74% of them were women. Mexican Americans make up 17.78% of the population. 37.00% of the population is between the ages of 20 and 40. The prevalences of low-eGFR, CKD, and albuminuria were 8.30%, 17.68%, and 12.16%, respectively, with an average SIRI of 1.20 ± 0.86. As shown in **Table 1**, the prevalence of low-eGFR, albuminuria, and CKD was significantly greater among people in the higher SIRI tertiles (all *p* < 0.05).

**Table 1:** Baseline characteristics according to SIRI tertiles.

SIRI	Overall	Tertile 1	Tertile 2	Tertile 3	*P-*value
		(0.06–0.79)	(0.79–1.27)	(1.27–24.60)	
N	41089	13650	13737	13702	
SIRI	1.20 ± 0.86	0.55 ± 0.15	1.01 ± 0.14	2.04 ± 1.01	<0.001
SII	537.07 ± 364.05	332.66 ± 146.24	493.21 ± 186.29	784.68 ± 486.29	<0.001
NHR	3.44 ± 2.03	2.31 ± 1.11	3.34 ± 1.42	4.67 ± 2.52	<0.001
MHR	0.45 ± 0.24	0.33 ± 0.20	0.44 ± 0.18	0.58 ± 0.26	<0.001
LHR	1.76 ± 1.07	1.82 ± 1.38	1.79 ± 0.86	1.68 ± 0.86	<0.001
PHR	200.85 ± 82.35	187.56 ± 74.02	201.83 ± 80.02	213.11 ± 90.19	<0.001
Age, years					<0.001
20-40	15203 (37.00%)	5422 (39.72%)	5222 (38.01%)	4559 (33.27%)	
41-60	14252 (34.69%)	5054 (37.03%)	4860 (35.38%)	4338 (31.66%)	
> 60	11634 (28.31%)	3174 (23.25%)	3655 (26.61%)	4805 (35.07%)	
Sex, n (%)					<0.001
Male	20451 (49.77%)	5913 (43.32%)	6764 (49.24%)	7774 (56.74%)	
Female	20638 (50.23%)	7737 (56.68%)	6973 (50.76%)	5928 (43.26%)	
Race, n (%)					<0.001
Mexican American	7307 (17.78%)	2316 (16.97%)	2602 (18.94%)	2389 (17.44%)	
Other Hispanic	3850 (9.37%)	1241 (9.09%)	1395 (10.16%)	1214 (8.86%)	
Non-Hispanic White	16678 (40.59%)	3726 (27.30%)	5904 (42.98%)	7048 (51.44%)	
Non-Hispanic Black	8915 (21.70%)	4623 (33.87%)	2382 (17.34%)	1910 (13.94%)	
Other Races	4339 (10.56%)	1744 (12.78%)	1454 (10.58%)	1141 (8.33%)	
Education level, n (%)					<0.001
Less than high school	10828 (26.35%)	3504 (25.67%)	3617 (26.33%)	3707 (27.05%)	
High school or GED	9512 (23.15%)	2959 (21.68%)	3105 (22.60%)	3448 (25.16%)	
Above high school	20707 (50.40%)	7175 (52.56%)	6998 (50.94%)	6534 (47.69%)	
Others	42 (0.10%)	12 (0.09%)	17 (0.12%)	13 (0.09%)	
Marital status, n (%)					<0.001
Married	17747 (52.46%)	5798 (52.10%)	6175 (54.05%)	5774 (51.21%)	
Never married	6287 (18.59%)	2233 (20.07%)	2012 (17.61%)	2042 (18.11%)	
Living with a partner	2606 (7.70%)	907 (8.15%)	881 (7.71%)	818 (7.25%)	
Others	7188 (21.25%)	2190 (19.68%)	2356 (20.62%)	2642 (23.43%)	
BMI, n (%)					<0.001
Normal weight	11879 (29.20%)	4347 (32.11%)	3871 (28.41%)	3661 (27.08%)	
Overweight	13566 (33.35%)	4570 (33.76%)	4627 (33.95%)	4369 (32.32%)	
Obese	15236 (37.45%)	4620 (34.13%)	5129 (37.64%)	5487 (40.59%)	
Smoking status, n (%)					< 0.001
≥100 cigarettes lifetime	18238 (44.42%)	5195 (38.09%)	5973 (43.50%)	7070 (51.65%)	
< 100 cigarettes lifetime	22820 (55.58%)	8444 (61.91%)	7759 (56.50%)	6617 (48.35%)	
PIR, n (%)					<0.001
Low income	13801 (37.04%)	4467 (36.08%)	4492 (36.15%)	4842 (38.87%)	
Medium income	12010 (32.23%)	3957 (31.96%)	3965 (31.91%)	4088 (32.82%)	
High income	11451 (30.73%)	3957 (31.96%)	3968 (31.94%)	3526 (28.31%)	
CVD, n (%)	3161 (7.69%)	640 (4.69%)	894 (6.51%)	1627 (11.87%)	< 0.001
Alcohol consumption, n (%)					<0.001
yes	25777 (78.67%)	8426 (79.78%)	8709 (79.41%)	8642 (76.91%)	
no	6988 (21.33%)	2135 (20.22%)	2258 (20.59%)	2595 (23.09%)	
Hypertension, n (%)	21512 (52.35%)	6527 (47.82%)	7005 (50.99%)	7980 (58.24%)	< 0.001
Diabetes, n (%)	6482 (15.78%)	1877 (13.75%)	1988 (14.47%)	2617 (19.10%)	< 0.001
TC, mg/dL	193.79 ± 41.36	195.23 ± 41.65	195.13 ± 40.66	191.01 ± 41.63	<0.001
ALT, U/L	25.45 ± 23.66	24.55 ± 17.38	25.64 ± 18.94	26.15 ± 31.89	<0.001
AST, U/L	25.36 ± 19.69	25.14 ± 15.03	25.11 ± 14.16	25.82 ± 27.14	0.004
Triglyceride, mg/dL	149.39 ± 119.98	138.11 ± 119.50	152.67 ± 117.86	157.34 ± 121.72	<0.001
Blood uric acid, mg/dL	5.44 ± 1.44	5.24 ± 1.37	5.43 ± 1.40	5.65 ± 1.52	<0.001
Serum phosphorus, mg/dL	3.69 ± 0.56	3.71 ± 0.55	3.69 ± 0.56	3.67 ± 0.57	<0.001
ACR, mg/g	48.01 ± 378.40	30.45 ± 263.45	38.80 ± 299.76	74.74 ± 518.73	<0.001
eGFR, mL/min/1.73 m2	92.32 ± 26.70	95.73 ± 25.54	92.45 ± 25.69	88.79 ± 28.32	<0.001
Low-eGFR, n (%)	3410 (8.30%)	683 (5.00%)	1019 (7.42%)	1708 (12.47%)	< 0.001
Albuminuria, n (%)	4997 (12.16%)	1293 (9.47%)	1458 (10.61%)	2246 (16.39%)	< 0.001
CKD, n (%)	7265 (17.68%)	1794 (13.14%)	2184 (15.90%)	3287 (23.99%)	< 0.001

SIRI, systemic inflammation response index; SII, systemic immune-inflammation index; NHR, neutrophil/high-density lipoprotein ratio; MHR, monocyte/high-density lipoprotein ratio; LHR, lymphocyte/high-density lipoprotein ratio; PHR, platelet/high-density lipoprotein ratio; GED, general educational development; BMI, body mass index; PIR, family income to poverty ratio; CVD, cardiovascular diseases; TC, total cholesterol; ALT, alanine aminotransferase; AST, aspartate aminotransferase; ACR, albumin-to-creatinine ratio; eGFR, estimated glomerular filtration rate; CKD, chronic kidney disease.

Numerous variables, such as sex, age, race, education level, BMI, smoking status, alcohol consumption, blood uric acid, serum phosphorus, hypertension, diabetes, TC, triglycerides, ALT, AST, PIR, CVD, marital status, ACR, eGFR, SII, NHR, LHR, MHR, and PHR, showed significant differences across SIRI tertiles (all *p* < 0.05).

### 3.2 Association between SIRI and CKD

**Table 2** displays the correlations between SIRI and other inflammatory biomarkers with CKD. According to the findings of our study, there is a positive link between SIRI, SII, NHR, and MHR with CKD in Models 1 and 2. After fully adjusting for covariates (Model 3), SIRI, SII, NHR, and MHR remain positively correlated with CKD (SIRI: OR = 1.24; 95% CI: 1.19, 1.30; SII: OR = 1.01; 95% CI: 1.01, 1.02; NHR: OR = 1.09; 95% CI: 1.06, 1.11; MHR: OR = 1.22; 95% CI: 1.03, 1.44). This implies that the prevalence of CKD rises by 24%, 1%, 9%, and 22%, respectively, with every unit increase in SIRI, SII, NHR, and MHR. In order to perform a sensitivity analysis, SIRI and other inflammatory biomarkers were divided into tertiles. People in the higher tertile of SIRI, SII, and NHR had a greater prevalence of CKD in Model 3 compared to participants in the lower tertile (all *p* for trend < 0.05).

**Table 2:** Associations between SIRI and other inflammatory biomarkers with CKD, albuminuria, and low-eGFR.

Index	Outcome	Continuous or categories	Model 1^3^		Model 2^4^		Model 3^5^	
			OR^1^ (95%CI^2^)	*P-* value	OR (95%CI)	*P-* value	OR (95%CI)	*P-* value
**SIRI**	**CKD**	SIRI as continuous variable	1.42 (1.38, 1.46)	<0.0001	1.33 (1.29, 1.37)	<0.0001	1.24 (1.19, 1.30)	<0.0001
		Tertile 1	Reference		Reference		Reference	
		Tertile 2	1.25 (1.17, 1.34)	<0.0001	1.24 (1.15, 1.33)	<0.0001	1.15 (1.03, 1.27)	0.0089
		Tertile 3	2.09 (1.96, 2.22)	<0.0001	1.88 (1.75, 2.02)	<0.0001	1.61 (1.45, 1.78)	<0.0001
		*P* for trend	<0.0001		<0.0001		<0.0001	
	**Albuminuria**	SIRI as continuous variable	1.34 (1.30, 1.38)	<0.0001	1.31 (1.27, 1.36)	<0.0001	1.27 (1.21, 1.32)	<0.0001
		Tertile 1	Reference		Reference		Reference	
		Tertile 2	1.13 (1.05, 1.23)	<0.0001	1.20 (1.11, 1.30)	<0.0001	1.16 (1.04, 1.30)	0.0109
		Tertile 3	1.87 (1.74, 2.02)	<0.0001	1.89 (1.75, 2.05)	<0.0001	1.73 (1.55, 1.94)	<0.0001
		*P* for trend	<0.0001		<0.0001		<0.0001	
	**Low-eGFR**	SIRI as continuous variable	1.44 (1.39, 1.48)	<0.0001	1.24 (1.20, 1.29)	<0.0001	1.11 (1.05, 1.18)	0.0003
		Tertile 1	Reference		Reference		Reference	
		Tertile 2	1.52 (1.38, 1.68)	0.0072	1.33 (1.19, 1.49)	<0.0001	1.11 (0.95, 1.30)	0.1898
		Tertile 3	2.70 (2.47, 2.96)	<0.0001	1.85 (1.66, 2.05)	<0.0001	1.34 (1.15, 1.56)	0.0002
		*P* for trend	<0.0001		<0.0001		<0.0001	
**SII**	**CKD**	SII as continuous variable	1.01 (1.01, 1.02)	<0.0001	1.01 (1.01, 1.02)	<0.0001	1.01 (1.01, 1.02)	<0.0001
		Tertile 1	Reference		Reference		Reference	
		Tertile 2	1.15 (1.08, 1.23)	<0.0001	1.22 (1.14, 1.31)	<0.0001	1.21 (1.10, 1.34)	0.0001
		Tertile 3	1.58 (1.48, 1.68)	<0.0001	1.66 (1.55, 1.77)	<0.0001	1.62 (1.47, 1.78)	<0.0001
		*P* for trend	<0.0001		<0.0001		<0.0001	
	**Albuminuria**	SII as continuous variable	1.01 (1.00, 1.01)	<0.0001	1.01 (1.01, 1.02)	<0.0001	1.01 (1.01, 1.02)	<0.0001
		Tertile 1	Reference		Reference		Reference	
		Tertile 2	1.11 (1.03, 1.20)	<0.0001	1.21 (1.12, 1.31)	<0.0001	1.24 (1.11, 1.39)	0.0001
		Tertile 3	1.58 (1.47, 1.70)	<0.0001	1.75 (1.62, 1.89)	<0.0001	1.74 (1.56, 1.93)	<0.0001
		*P* for trend	<0.0001		<0.0001		<0.0001	
	**Low-eGFR**	SII as continuous variable	1.01 (1.00, 1.01)	<0.0001	1.01 (1.01, 1.02)	<0.0001	1.01 (1.01, 1.02)	0.0028
		Tertile 1	Reference		Reference		Reference	
		Tertile 2	1.21 (1.10, 1.32)	<0.0001	1.22 (1.10, 1.35)	0.0001	1.19 (1.03, 1.38)	0.0178
		Tertile 3	1.58 (1.45, 1.72)	<0.0001	1.46 (1.32, 1.60)	<0.0001	1.34 (1.16, 1.54)	<0.0001
		*P* for trend	<0.0001		<0.0001		<0.0001	
**NHR**	**CKD**	NHR as continuous variable	1.10 (1.09, 1.12)	<0.0001	1.19 (1.17, 1.21)	<0.0001	1.09 (1.06, 1.11)	<0.0001
		Tertile 1	Reference		Reference		Reference	
		Tertile 2	1.28 (1.20, 1.36)	<0.0001	1.44 (1.35, 1.55)	<0.0001	1.23 (1.12, 1.36)	<0.0001
		Tertile 3	1.57 (1.47, 1.67)	<0.0001	2.14 (1.99, 2.30)	<0.0001	1.47 (1.32, 1.63)	<0.0001
		*P* for trend	<0.0001		<0.0001		<0.0001	
	**Albuminuria**	NHR as continuous variable	1.12 (1.10, 1.14)	<0.0001	1.18 (1.16, 1.20)	<0.0001	1.08 (1.06, 1.11)	<0.0001
		Tertile 1	Reference		Reference		Reference	
		Tertile 2	1.26 (1.16, 1.36)	<0.0001	1.39 (1.28, 1.50)	<0.0001	1.22 (1.09, 1.37)	0.0007
		Tertile 3	1.68 (1.56, 1.81)	<0.0001	2.12 (1.96, 2.30)	<0.0001	1.55 (1.37, 1.75)	<0.0001
		*P* for trend	<0.0001		<0.0001		<0.0001	
	**Low-eGFR**	NHR as continuous variable	1.07 (1.05, 1.08)	<0.0001	1.16 (1.14, 1.19)	<0.0001	1.02 (0.99, 1.06)	0.1082
		Tertile 1	Reference		Reference		Reference	
		Tertile 2	1.42 (1.29, 1.55)	<0.0001	1.61 (1.46, 1.78)	<0.0001	1.28 (1.11, 1.49)	0.0009
		Tertile 3	1.52 (1.40, 1.67)	<0.0001	2.20 (1.98, 2.43)	<0.0001	1.35 (1.14, 1.58)	0.0003
		*P* for trend	<0.0001		<0.0001		0.0012	
**MHR**	**CKD**	MHR as continuous variable	1.99 (1.79, 2.21)	<0.0001	2.63 (2.34, 2.97)	<0.0001	1.22 (1.03, 1.44)	0.0248
		Tertile 1	Reference		Reference		Reference	
		Tertile 2	1.15 (1.08, 1.23)	<0.0001	1.26 (1.17, 1.35)	<0.0001	1.03 (0.93, 1.14)	0.5635
		Tertile 3	1.47 (1.38, 1.56)	<0.0001	1.73 (1.61, 1.85)	<0.0001	1.12 (1.01, 1.25)	0.0357
		*P* for trend	<0.0001		<0.0001		0.0273	
	**Albuminuria**	MHR as continuous variable	1.85 (1.65, 2.08)	0.0071	2.22 (1.95, 2.52)	0.0073	1.21 (1.03, 1.44)	0.0239
		Tertile 1	Reference		Reference		Reference	
		Tertile 2	1.11 (1.03, 1.19)	0.0092	1.17 (1.08, 1.27)	<0.0001	0.98 (0.88, 1.09)	0.7032
		Tertile 3	1.44 (1.34, 1.55)	<0.0001	1.61 (1.49, 1.75)	<0.0001	1.12 (1.00, 1.26)	0.0548
		*P* for trend	<0.0001		<0.0001		0.0250	
	**Low-eGFR**	MHR as continuous variable	2.18 (1.91, 2.49)	<0.0001	2.74 (2.33, 3.22)	<0.0001	1.09 (0.88, 1.35)	0.4275
		Tertile 1	Reference		Reference		Reference	
		Tertile 2	1.25 (1.14, 1.37)	<0.0001	1.35 (1.22, 1.49)	<0.0001	1.07 (0.93, 1.24)	0.3427
		Tertile 3	1.63 (1.49, 1.78)	<0.0001	1.90 (1.72, 2.11)	<0.0001	1.09 (0.93, 1.28)	0.2773
		*P* for trend	<0.0001		<0.0001		0.3224	
**LHR**	**CKD**	LHR as continuous variable	0.97 (0.95, 1.00)	0.0505	1.10 (1.07, 1.13)	<0.0001	0.97 (0.94, 1.01)	0.1485
		Tertile 1	Reference		Reference		Reference	
		Tertile 2	0.79 (0.75, 0.84)	<0.0001	1.02 (0.96, 1.09)	0.4973	0.87 (0.79, 0.96)	0.0047
		Tertile 3	0.84 (0.79, 0.90)	<0.0001	1.29 (1.21, 1.39)	<0.0001	0.83 (0.75, 0.93)	0.0008
		*P* for trend	<0.0001		<0.0001		0.0014	
	**Albuminuria**	LHR as continuous variable	1.01 (0.98, 1.03)	0.6697	1.06 (1.03, 1.09)	<0.0001	0.95 (0.91, 1.01)	0.0507
		Tertile 1	Reference		Reference		Reference	
		Tertile 2	0.84 (0.78, 0.91)	<0.0001	0.98 (0.91, 1.06)	0.6055	0.83 (0.74, 0.92)	0.0006
		Tertile 3	0.98 (0.91, 1.05)	0.5255	1.25 (1.16, 1.35)	<0.0001	0.82 (0.73, 0.93)	0.0012
		*P* for trend	0.9622		<0.0001		0.0031	
	**Low-eGFR**	LHR as continuous variable	0.87 (0.84, 0.91)	<0.0001	1.07 (1.03, 1.11)	0.0002	0.98 (0.95, 1.02)	0.4164
		Tertile 1	Reference		Reference		Reference	
		Tertile 2	0.70 (0.65, 0.77)	<0.0001	1.05 (0.96, 1.15)	0.3173	0.91 (0.79, 1.04)	0.1514
		Tertile 3	0.65 (0.60, 0.71)	<0.0001	1.30 (1.18, 1.43)	<0.0001	0.82 (0.71, 0.96)	0.0144
		*P* for trend	<0.0001		<0.0001		0.0150	
**PHR**	**CKD**	PHR as continuous variable	1.00 (0.98, 1.01)	0.8444	1.01 (1.00, 1.01)	<0.0001	1.01 (1.00, 1.01)	0.0373
		Tertile 1	Reference		Reference		Reference	
		Tertile 2	0.88 (0.83, 0.94)	0.0001	1.08 (1.01, 1.16)	0.0219	0.97 (0.89, 1.07)	0.5930
		Tertile 3	0.96 (0.90, 1.02)	0.2206	1.49 (1.39, 1.59)	<0.0001	1.06 (0.95, 1.17)	0.3037
		*P* for trend	0.3859		<0.0001		0.2480	
	**Albuminuria**	PHR as continuous variable	1.01 (1.00, 1.01)	<0.0001	1.01 (1.00, 1.01)	<0.0001	1.01 (1.00, 1.01)	0.0031
		Tertile 1	Reference		Reference		Reference	
		Tertile 2	0.90 (0.84, 0.97)	0.0054	1.03 (0.95, 1.11)	0.4857	0.91 (0.81, 1.01)	0.0723
		Tertile 3	1.13 (1.05, 1.21)	0.0011	1.49 (1.39, 1.61)	<0.0001	1.09 (0.97, 1.22)	0.1377
		*P* for trend	0.0001		<0.0001		0.0618	
	**Low-eGFR**	PHR as continuous variable	0.99 (0.98, 0.99)	<0.0001	1.01 (1.00, 1.01)	<0.0001	0.99 (0.99, 1.01)	0.4689
		Tertile 1	Reference		Reference		Reference	
		Tertile 2	0.87 (0.80, 0.95)	0.0014	1.20 (1.09, 1.31)	0.2229	1.09 (0.95, 1.25)	0.2236
		Tertile 3	0.76 (0.69, 0.82)	<0.0001	1.47 (1.33, 1.62)	<0.0001	0.99 (0.85, 1.15)	0.8986
		*P* for trend	<0.0001		<0.0001		0.8323	

In sensitivity analysis, SIRI, SII, NHR, MHR, LHR, and PHR were converted from continuous variables to categorical variables (tertiles).

^1^OR: Odd ratio.

^2^95% CI: 95% confidence interval.

^3^Model 1: No covariates were adjusted.

^4^Model 2: Adjusted for age, sex, and race.

^5^Model 3: Adjusted for sex, age, race, education level, BMI, smoking status, alcohol consumption, blood uric acid, serum phosphorus, TC, triglycerides, AST, ALT, PIR, CVD, marital status, diabetes, and hypertension.

**Figure 2:**
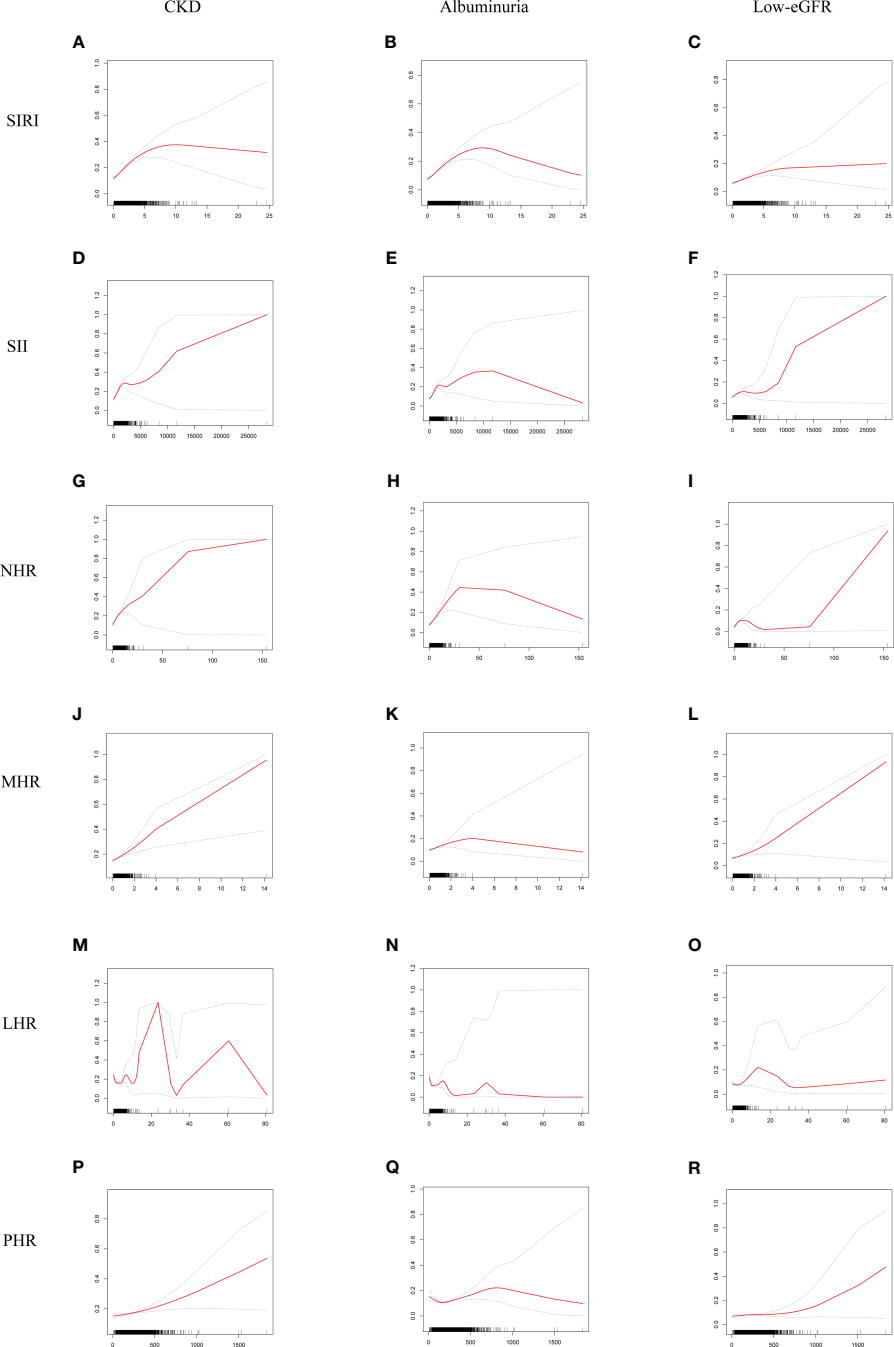
Smooth curve fitting for SIRI and other inflammatory biomarkers with CKD, albuminuria, and low-eGFR. **(A)** SIRI and CKD; **(B)** SIRI and albuminuria; **(C)** SIRI and low-eGFR; **(D)** SII and CKD; **(E)** SII and albuminuria; **(F)** SII and low-eGFR; **(G)** NHR and CKD; **(H)** NHR and albuminuria; **(I)** NHR and low-eGFR; **(J)** MHR and CKD; **(K)** MHR and albuminuria; **(L)** MHR and low-eGFR; **(M)** LHR and CKD; **(N)** LHR and albuminuria; **(O)** LHR and low-eGFR; **(P)** PHR and CKD; **(Q)** PHR and albuminuria; **(R)** PHR and low-eGFR.

Smooth curve fitting and GAM indicated that the relationships between SIRI, SII, NHR, LHR, and PHR with CKD were nonlinear (**Figure 2**). After full adjustment, breakpoints (K) were found to be 2.04, 2105.48, 3.37, 1.2, and 113.14, respectively (all logarithmic likelihood ratio test *P*-value <0.05). SIRI and CKD are both positively correlated on the two sides of the breakpoint (**Table 3**).

**Table 3:** Threshold effect analysis of SIRI and other inflammatory biomarkers on CKD, albuminuria, and low-eGFR using a two-piecewise linear regression model in Model 3.

	CKD		Albuminuria		Low-eGFR	
	OR^1^ (95%CI^2^)	*P-* value	OR (95%CI)	*P-* value	OR (95%CI)	*P-* value
SIRI						
**Fitting by standard linear model**	1.24 (1.19, 1.30)	<0.0001	1.27 (1.21, 1.32)	<0.0001	1.11 (1.05, 1.18)	0.0003
Fitting by two-piecewise linear model						
Breakpoint (K)	2.04		2.18		1.85	
OR1(< K)	1.47 (1.36, 1.60)	<0.0001	1.55 (1.43, 1.68)	<0.0001	1.29 (1.14, 1.47)	0.0001
OR2(> K)	1.09 (1.01, 1.16)	0.0174	1.08 (1.01, 1.16)	0.0188	1.03 (0.95, 1.11)	0.4401
OR2/OR1	0.74 (0.65, 0.83)	<0.0001	0.70 (0.62, 0.79)	<0.0001	0.80 (0.67, 0.95)	0.0099
Logarithmic likelihood ratio test P-value	<0.001		<0.001		0.011	
SII						
Fitting by standard linear model	1.01 (1.01, 1.02)	<0.0001	1.01 (1.01, 1.02)	<0.0001	1.01 (1.01, 1.02)	0.0028
Fitting by two-piecewise linear model						
Breakpoint (K)	2105.48		1924.3		682.5	
OR1(< K)	1.01 (1.00, 1.02)	<0.0001	1.01 (1.01, 1.02)	<0.0001	1.01 (1.00, 1.02)	<0.0001
OR2(> K)	0.99 (0.99, 1.01)	0.7294	0.99 (0.98, 1.01)	0.3143	0.99 (0.98, 1.01)	0.6353
OR2/OR1	1.01 (1.00, 1.02)	<0.0001	1.01 (1.01, 1.02)	<0.0001	1.00 (1.00, 1.01)	0.0008
Logarithmic likelihood ratio test P-value	<0.001		<0.001		0.001	
NHR						
Fitting by standard linear model	1.09 (1.06, 1.11)	<0.0001	1.08 (1.06, 1.11)	<0.0001	1.02 (0.99, 1.06)	0.1082
Fitting by two-piecewise linear model						
Breakpoint (K)	3.37		5.96		3.31	
OR1(< K)	1.19 (1.12, 1.27)	<0.0001	1.16 (1.12, 1.20)	<0.0001	1.22 (1.11, 1.35)	<0.0001
OR2(> K)	1.06 (1.03, 1.09)	<0.0001	1.01 (0.99, 1.03)	0.3451	1.00 (0.98, 1.02)	0.9676
OR2/OR1	0.89 (0.83, 0.96)	0.0029	0.87 (0.84, 0.91)	<0.0001	0.82 (0.74, 0.90)	<0.0001
Logarithmic likelihood ratio test P-value	0.003		<0.001		<0.001	
MHR						
Fitting by standard linear model	1.22 (1.03, 1.44)	0.0248	1.21 (1.03, 1.44)	0.0239	1.09 (0.88, 1.35)	0.4275
Fitting by two-piecewise linear model						
Breakpoint (K)	0.25		0.87		0.19	
OR1(< K)	0.65 (0.12, 3.59)	0.6231	1.48 (1.15, 1.92)	0.0027	0.02 (0.01, 2.04)	0.0899
OR2(> K)	1.24 (1.04, 1.48)	0.0195	0.99 (0.74, 1.33)	0.9495	1.12 (0.90, 1.40)	0.3212
OR2/OR1	1.90 (0.33, 10.91)	0.4716	0.67 (0.44, 1.03)	0.0651	109.75 (0.53, 226.05)	0.0840
Logarithmic likelihood ratio test P-value	0.473		0.047		0.092	
LHR						
Fitting by standard linear model	0.97 (0.94, 1.01)	0.1485	0.95 (0.91, 1.01)	0.0507	0.98 (0.95, 1.02)	0.4164
Fitting by two-piecewise linear model						
Breakpoint (K)	1.2		1.19		1.88	
OR1(< K)	0.55 (0.43, 0.68)	<0.0001	0.48 (0.37, 0.62)	<0.0001	0.79 (0.68, 0.91)	0.0016
OR2(> K)	1.00 (0.97, 1.03)	0.9485	0.99 (0.96, 1.03)	0.7460	1.01 (0.97, 1.04)	0.7245
OR2/OR1	1.83 (1.45, 2.32)	<0.0001	2.07 (1.58, 2.71)	<0.0001	1.27 (1.09, 1.49)	0.0022
Logarithmic likelihood ratio test P-value	<0.001		<0.001		0.002	
PHR						
Fitting by standard linear model	1.01 (1.00, 1.01)	0.0373	1.01 (1.00, 1.01)	0.0031	0.99 (0.99, 1.01)	0.4689
Fitting by two-piecewise linear model						
Breakpoint (K)	113.14		153.3		178.07	
OR1(< K)	0.99 (0.99, 1.00)	0.0229	0.98 (0.98, 0.99)	0.0010	1.00 (0.99, 1.01)	0.2149
OR2(> K)	1.00 (1.00, 1.01)	0.0055	1.00 (1.00, 1.01)	<0.0001	1.00 (0.99, 1.01)	0.1211
OR2/OR1	1.01 (1.00, 1.01)	0.0119	1.00 (1.00, 1.01)	<0.0001	0.99 (0.99, 1.01)	0.1089
Logarithmic likelihood ratio test P-value	0.013		<0.001		0.107	

Adjusted for sex, age, race, education level, BMI, smoking status, alcohol consumption, blood uric acid, serum phosphorus, TC, triglycerides, AST, ALT, PIR, CVD, marital status, diabetes, and hypertension.

^1^OR: Odd ratio.

^2^95% CI: 95% confidence interval.

### 3.3 Association between SIRI and albuminuria

Additionally, we discovered that the prevalence of albuminuria rises along with levels of SIRI, SII, NHR, MHR, and PHR. For every unit increase in SIRI, SII, NHR, MHR, and PHR, the prevalences of albuminuria increase by 27%, 1%, 8%, 21%, and 1% in Model 3 (SIRI: OR = 1.27; 95% CI: 1.21, 1.32; SII: OR = 1.01; 95% CI: 1.01, 1.02; NHR: OR = 1.08; 95% CI: 1.06, 1.11; MHR: OR = 1.21; 95% CI: 1.03, 1.44; PHR: OR = 1.01; 95% CI: 1.00, 1.01). There are still substantial correlations even once these inflammatory biomarkers are divided into tertiles. As compared to those in the lower tertile, the higher tertiles of SIRI, SII, and NHR persons in Model 3 demonstrated a higher prevalence of albuminuria (**Table 2**).

SIRI, SII, NHR, MHR, LHR, and PHR showed nonlinear associations with albuminuria, according to the GAM and smooth curve fitting. Breakpoints after complete adjustment were discovered to be 2.18, 1924.3, 5.96, 0.87, 1.19, and 153.3, respectively. SIRI and albuminuria are positively correlated on the two sides of the breakpoint (**Table 3**).

### 3.4 Association between SIRI and low-eGFR

We also assessed the relationships between SIRI and other inflammatory biomarkers with low-eGFR using three different models (**Table 2**). In the fully adjusted model, low-eGFR was strongly correlated with SIRI and SII (SIRI: OR = 1.11; 95% CI: 1.05, 1.18; SII: OR = 1.01; 95% CI: 1.01, 1.02). Those in the tertiles with the highest SIRI had the highest prevalence of low-eGFR, as compared to those in the lowest tertiles (*p* for trend < 0.05).

GAM and smooth curve fitting indicated that the relationships between SIRI, SII, NHR, and LHR with low-eGFR were nonlinear (**Figure 2**). Breakpoints after complete adjustment were discovered to be 1.85, 682.5, 3.31, and 1.88, respectively. In the nonlinear relationship between SIRI and low-eGFR, we also noticed a saturation effect. SIRI and the prevalence of low-eGFR are positively correlated when SIRI is less than 1.85 (OR = 1.29; 95% CI: 1.14, 1.47). The two do not significantly correlate on the right side of the breakpoint (OR = 1.03; 95% CI: 0.95, 1.11) (**Table 3**).

### 3.5 Subgroup analysis

**Figure 3:**
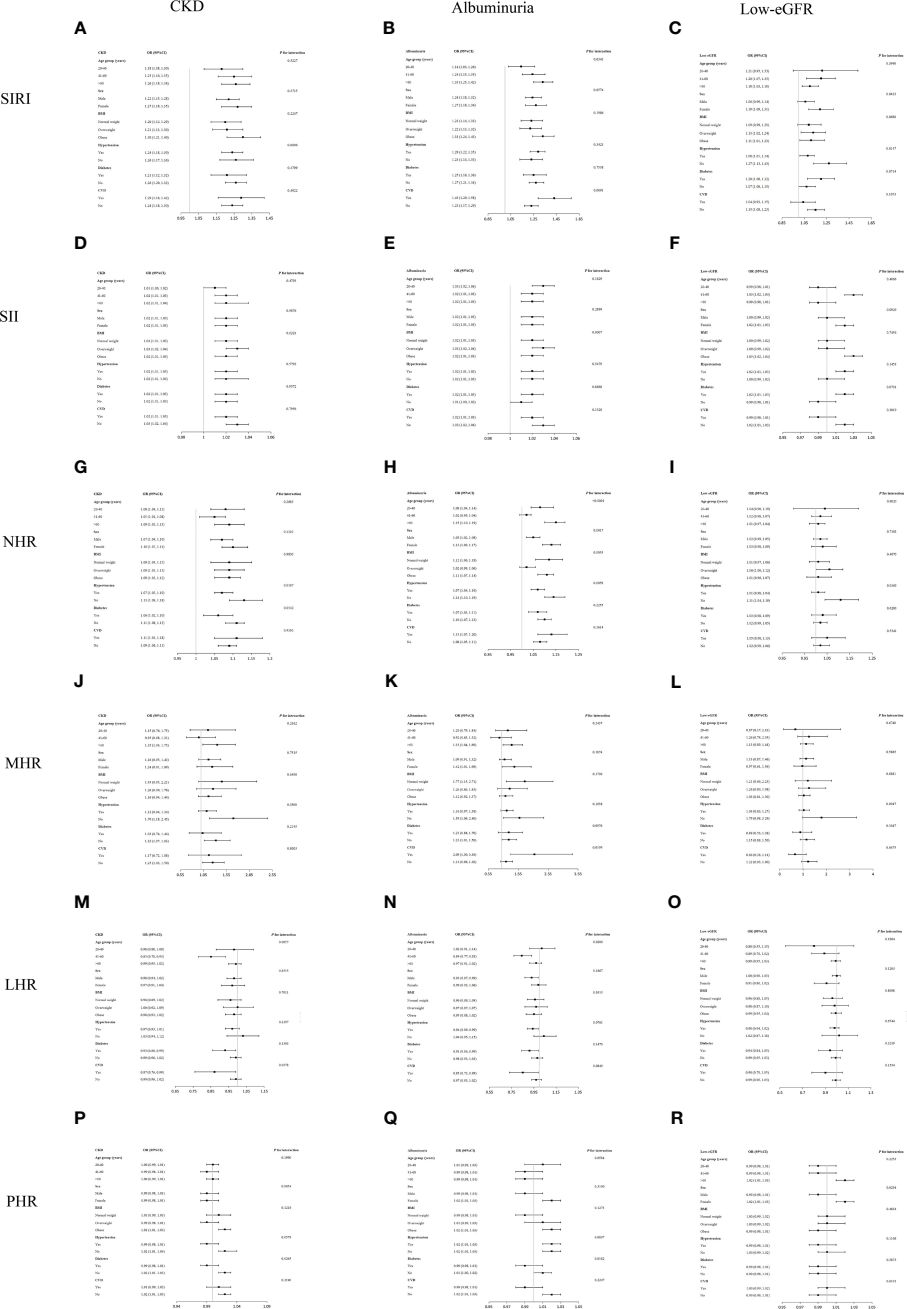
Subgroup analysis for the association of SIRI and other inflammatory biomarkers with CKD, albuminuria, and low-eGFR. **(A)** SIRI and CKD; **(B)** SIRI and albuminuria; **(C)** SIRI and low-eGFR; **(D)** SII and CKD; **(E)** SII and albuminuria; **(F)** SII and low-eGFR; **(G)** NHR and CKD; **(H)** NHR and albuminuria; **(I)** NHR and low-eGFR; **(J)** MHR and CKD; **(K)** MHR and albuminuria; **(L)** MHR and low-eGFR; **(M)** LHR and CKD; **(N)** LHR and albuminuria; **(O)** LHR and low-eGFR; **(P)** PHR and CKD; **(Q)** PHR and albuminuria; **(R)** PHR and low-eGFR.

Subgroup analysis showed that SIRI and CKD were positively correlated in all groupings (**Figure 3**). The relationships between SIRI and MHR with CKD were not substantially associated in the interaction tests for the different strata, suggesting that this positive association was the same across populations(all *p* for interaction > 0.05).

An analysis of the interaction test revealed that no stratum showed a significant effect of sex, BMI, diabetes, or hypertension on the relationships between SIRI and albuminuria (**Figure 3**). The association between MHR and albuminuria showed a dependence on CVD status, which may be applicable to CVD patients. The association between PHR and albuminuria showed a dependence on diabetes and may apply to a non-diabetic US population.

Per the interaction test, age, BMI, diabetes, or CVD subgroups did not substantially affect the relationship between SIRI and low-eGFR (*p* for interaction > 0.05). The association of NHR with low-eGFR showed dependence on hypertension status and may apply to non-hypertension populations. The association of PHR with albuminuria showed dependence on sex and may apply to female populations (**Figure 3**).

### 3.6 ROC analysis

**Figure 4:**
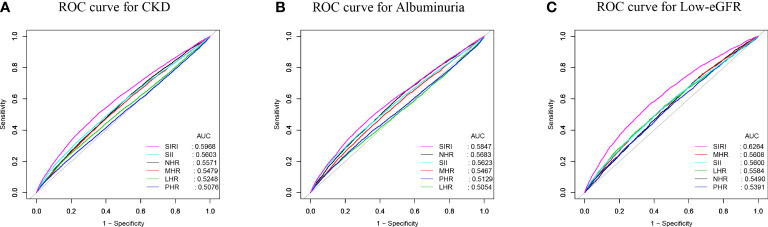
ROC curves and the AUC values of the six inflammatory biomarkers (SIRI, SII, NHR, PHR, MHR, and LHR) in diagnosing CKD, albuminuria and low-eGFR. **(A)** Six obesity indicators were assessed to identify CKD. **(B)** Six obesity indicators were assessed to identify albuminuria. **(C)** Six obesity indicators were assessed to identify low-eGFR.

To evaluate SIRI’s predictive power for CKD, albuminuria, and low-eGFR against other inflammatory biomarkers (SII, NHR, LHR, MHR, and PHR), we computed the AUC values (**Figure 4**). Our results show that SIRI had higher AUC values than the other five inflammatory biomarkers in terms of predicting CKD, albuminuria, and low-eGFR. Additionally, **Table 4** demonstrates that AUC values for SIRI and the other inflammatory biomarkers differed statistically significantly (all *p* < 0.05). These results indicate that, in comparison to other inflammatory biomarkers (SII, NHR, LHR, MHR, and PHR), SIRI has the best discriminative ability and accuracy in predicting CKD, albuminuria, and low-eGFR.

**Table 4:** Comparison of AUC values between SIRI and other inflammatory biomarkers.

Test	AUC^1^	95%CI^2^ low	95%CI upp	Best threshold	Specificity	Sensitivity	*P* for different in AUC
CKD							
**SIRI**	0.5968	0.5894	0.6041	1.1741	0.6382	0.5120	Reference
**SII**	0.5603	0.5528	0.5678	606.0263	0.7167	0.3747	<0.0001
**NHR**	0.5571	0.5498	0.5644	2.8219	0.4539	0.6312	<0.0001
**MHR**	0.5479	0.5405	0.5552	0.4336	0.5718	0.5026	<0.0001
**LHR**	0.5173	0.5324	1.0537	0.8141	0.2399	0.5173	<0.0001
**PHR**	0.5076	0.5001	0.5151	129.1092	0.8350	0.1937	<0.0001
Albuminuria							
**SIRI**	0.5847	0.5760	0.5934	1.2465	0.6688	0.4659	Reference
**SII**	0.5623	0.5535	0.5711	620.0719	0.7285	0.3696	<0.0001
**NHR**	0.5683	0.5597	0.5769	3.0242	0.5045	0.5980	<0.0001
**MHR**	0.5467	0.5380	0.5554	0.4399	0.5842	0.4905	<0.0001
**LHR**	0.5054	0.4965	0.5143	0.9246	0.8746	0.1594	<0.0001
**PHR**	0.5129	0.5040	0.5219	228.7314	0.7059	0.3361	<0.0001
Low-eGFR							
**SIRI**	0.6264	0.6165	0.6364	1.1771	0.6305	0.5601	Reference
**SII**	0.5600	0.5497	0.5704	501.7929	0.5668	0.5270	<0.0001
**NHR**	0.5490	0.5391	0.5588	2.4417	0.3417	0.7397	<0.0001
**MHR**	0.5608	0.5507	0.5710	0.4592	0.6184	0.4736	<0.0001
**LHR**	0.5584	0.5480	0.5688	1.2411	0.7055	0.3908	<0.0001
**PHR**	0.5391	0.5289	0.5493	190.5980	0.4868	0.5748	<0.0001

^1^AUC, area under the curve.

^2^95% CI, 95% confidence interval.

## 4 Discussion

This cross-sectional study of 41,089 adult participants revealed a positive correlation between the prevalence of CKD and SIRI. Using smooth curve fitting, we also found a nonlinear connection between SIRI and CKD, with breakpoints set at 2.04. Additionally, it was discovered that low-eGFR and albuminuria have positive and nonlinear correlations with SIRI, with breakpoints at 1.85 and 2.18, respectively. Subgroup studies and interaction testing revealed no statistically significant variation in the relationship between SIRI and CKD between the groups. Additional inflammatory biomarkers and CKD were linked, as we also saw. There were correlations between greater levels of SII, NHR, MHR, and PHR with a higher prevalence of CKD. When SIRI is compared against SII, NHR, LHR, MHR, and PHR, among other inflammatory biomarkers, ROC analysis indicates that SIRI might be a more accurate indicator of low-eGFR, CKD, and albuminuria. Finally, given the significance of the high SIRI values in evaluating kidney health in adult Americans, it is important to emphasize them.

The primary focus of our research was the association between renal function and other inflammatory indicators and SIRI. According to our research, high levels of MHR and NHR are linked to higher prevalences of CKD and albuminuria. This association can be attributed to the fact that these inflammatory biomarkers are novel biomarkers related to whole blood cells and HDL-C. Inflammation can lead to changes in whole blood cells, including neutrophils, monocytes, and platelets. In addition to its function in transporting cholesterol, HDL-C has a number of anti-infection, anti-inflammatory, antioxidant, and anti-thrombotic characteristics (22). Similar to our study, Prior studies have discovered predictive significance for MHR and NHR in conditions such as peripheral arterial disease (PAD), acute myocardial infarction, and schizophrenia with concomitant bipolar affective disorder (23–25). In earlier research, the relationship between SII and renal function was also examined. SII and albuminuria have been shown to be positively correlated, according to Qin et al.’s cross-sectional research of 36,463 adult Americans (7). In Chinese CKD patients, Lai et al. discovered that SII is an independent risk factor for all-cause, cardiovascular, and cancer-related death (26). Our study found that for every unit rise in SII, the prevalence of CKD, albuminuria, and low-eGFR increased by 1%. Because of this, SII, a novel inflammation biomarker that integrates platelets, neutrophils, and lymphocytes, can forecast kidney function in adult Americans. These inflammatory biomarkers (SII, NHR, and MHR) were not the best biomarkers for predicting kidney function, according to ROC analysis, as they had significantly lower AUC values. To support our conclusions, more prospective research is required.

The key conclusion of this study, which to our knowledge is the first to examine the connection between SIRI and CKD, is that there is a positive correlation. In earlier studies, the relationship between SIRI and other illnesses was mainly examined. The sickness severity of acute pancreatitis (AP) and development of acute kidney damage (AKI) have both been found to be predicted by SIRI (4). The all-cause and CVD deaths in adult Americans have also been strongly correlated with it (6). Preoperative SIRI, according to Lv et al.’s research, is a reliable indicator of postoperative prognosis in patients with renal cell carcinoma and inferior vena cava tumor thrombus (RCC-IVCTT) (27). The prevalence of CKD rose by 24% in our sample, with each unit rising in SIRI. We also identified a nonlinear association between SIRI and CKD. SIRI exhibited the positive connections with CKD in the two sides of the breakpoint (SIRI = 2.04). This means that the higher the SIRI level, the greater the threat to kidney health. The prevalence of albuminuria and low-eGFR were also observed to be positively correlated with an increase in SIRI. Additionally, there were nonlinear connections between SIRI with albuminuria and low-eGFR, with breakpoints at 2.18 and 1.85, respectively. In conclusion, adult Americans’ renal function is significantly impacted negatively by SIRI.

The association between the two may be attributed to the fact that SIRI is a novel systemic inflammatory biomarker based on the counts of neutrophils, monocytes, and lymphocytes in peripheral blood. Monocytes can contribute to this inflammatory response by releasing pro-inflammatory cytokines and interacting with other immune cells such as lymphocytes and neutrophils. Renal fibrosis has also been linked to dysregulation of the monocyte-derived transforming growth factor-beta (TGF-β) (28, 29). Through the release of several pro-inflammatory mediators and the production of reactive oxygen species (ROS), neutrophils have a role in the pathophysiology of CKD (30). A substantial correlation has been discovered between lymphocytes, particularly the decline of CD4 T lymphocytes, and worsening kidney function (31). The superiority of SIRI in predicting other diseases has also been investigated in earlier research. The highest AUC value was achieved by SIRI in the prediction of peripheral arterial disease (PAD) in patients with T2DM when compared to AISI, SII, NHR, MHR, and PHR (25). In the context of cervical cancer prognostic prediction, SIRI demonstrated superior accuracy in comparison to NLR, PLR, and MLR (32). SIRI beat NLR, PLR, lymphocyte-to-monocyte ratio (LMR), and red blood cell distribution width (RDW) for predicting the prognosis of stroke (33). Furthermore, in comparison to other inflammation biomarkers (SII, NHR, LHR, MHR, and PHR), our research shows that SIRI may have a better discriminative capacity and accuracy in predicting CKD, albuminuria, and low-eGFR. Because of its cost-effectiveness and accessibility, SIRI can be seen as a more accurate and complete inflammatory biomarker. The ability of SIRI to evaluate kidney health in adult Americans has enormous potential, to sum up.

CKD has a number of important risk factors, including CVD (3). This opinion is backed up by our research. This is supported by our study, where subgroup analyses showed that the prevalence of CKD was higher in the CVD population than in the non-CVD population for each unit increase in SIRI. Subgroup analysis also reveals that obese people are more likely to develop CKD than normal-weight or overweight people, probably as a result of obesity’s impact on the kidneys through inflammation and insulin resistance (34). Furthermore, our results show that age, sex, BMI, hypertension, diabetes, and CVD had no appreciable influence on the association between SIRI with CKD. These study findings add to the body of evidence demonstrating SIRI’s detrimental impact on renal health.

It is still unknown what the underlying mechanisms are that connect SIRI to CKD. A number of tubular toxins, such as ROS, are produced in response to systemic or intrarenal inflammation. These toxins lead to tubular damage, nephron dropout, and the start of chronic kidney disease. Proinflammatory cytokines in circulation activate leukocytes and endothelial cells found in intrarenal microvessels, leading to a localized rise in proinflammatory factors and ROS. These mechanisms induce disruptions in the glycocalyx layer and affect the cell-surface adhesion molecules. Receptor-mediated vasoreactivity, endothelial barrier function, and the activation of the coagulation system are also compromised. These inflammatory-induced alterations may result in irreversible tubular damage and nephron failure (35, 36).

There are advantages to our study. Firstly, the sample selection was representative, with a sufficiently large sample size. Second, in order to get more accurate results, we also made adjustments for confounders. But because of a number of restrictions, the study’s findings should be regarded cautiously. In the first place, we were unable to determine causal linkages because of the cross-sectional study design. Therefore, to clarify causality, prospective research with bigger sample numbers is still required. Second, even after adjusting for a few potential factors, the impacts of additional potential confounders could not be fully ruled out. Thirdly, Median imputation, which is unaffected by outliers, can better maintain the overall trend and distribution pattern of the data, but cannot fully utilize all the information in the data set. Though it is a good approximation of the ground truth, mode imputation—especially for skewed distributions—can add bias by substituting the most common value for missing values (37, 38). In conclusion, our study presents important strengths but is not without limitations. Careful consideration of these limitations is necessary when interpreting our findings. Further research, particularly prospective studies with diverse populations, is needed to confirm and expand upon our results.

## 5 Conclusions

When predicting CKD, albuminuria, and low-eGFR, SIRI may show up as a superior inflammatory biomarker when compared to other inflammatory biomarkers (SII, NHR, LHR, MHR, and PHR). American adults with elevated levels of SIRI, SII, NHR, MHR, and PHR should be attentive to the potential risks to their kidney health.

## Data availability statement

Publicly available datasets were analyzed in this study. This data can be found here: https://www.cdc.gov/nchs/nhanes.

## Ethics statement

The studies involving humans were approved by The National Center for Health Statistics Ethics Review Board. The studies were conducted in accordance with the local legislation and institutional requirements. Written informed consent for participation was not required from the participants or the participants’ legal guardians/next of kin in accordance with the national legislation and institutional requirements. Written informed consent was obtained from the individual(s) for the publication of any potentially identifiable images or data included in this article.

## Author contributions

XL: Writing – original draft. LC: Writing – review & editing. HX: Conceptualization, Writing – review & editing.
